# Presence of kratom in opioid overdose deaths: findings from coroner postmortem toxicological report

**DOI:** 10.3389/fpsyt.2023.1332999

**Published:** 2024-01-10

**Authors:** Tyler Torrico, Kajal Patel, Nicole Nikolov, Md. Towhid Salam, Ranjit Padhy, David Weinstein

**Affiliations:** ^1^Department of Psychiatry, Kern Medical, Bakersfield, CA, United States; ^2^Ross University School of Medicine, Miramar, FL, United States; ^3^Department of Population and Public Health Sciences, University of Southern California, Los Angeles, CA, United States

**Keywords:** accidental overdose, drug abuse, mitragynine, opioid, stimulant, substance abuse

## Abstract

**Background:**

Kratom (*Mitragyna speciosa*) use in the United States is becoming increasingly popular and its legal status varies widely from state to state. Multiple reports of adverse events associated with kratom use have ranged from liver injury, seizures, psychiatric disturbance, and rarely death.

**Methods:**

This study investigated coroner autopsy reports from Kern County in California for the year 2020 which included qualitative data on substances from blood toxicological reports. Of the 214 opioid-associated accidental overdoses reported, 4 subjects (1.9%) had mitragynine (kratom) exposure on the autopsy report and were included in the study. We reported available demographic information and comorbid substance findings from the associated autopsy reports.

**Results:**

All 4 individuals with mitragynine (kratom) toxicology had accidental opioid overdose deaths noted in autopsy reports. Each subject also had toxicology positive for at least one other substance. Fentanyl was found in 3 (75%) of the cases and suspected to be the primary contributor to opioid-related deaths in those cases. However, one fatality was without fentanyl, but instead had tested positive for benzodiazepines, cannabis, and other psychiatric medications.

**Discussion:**

The findings of this brief report provide insight into the role that mitragynine (kratom) may have in modulating risk of opioid-related deaths. The combined use of kratom with opioids such as fentanyl appears most likely to increase the risk of a fatal overdose, but it may also occur with other medications such as benzodiazepines and psychiatric medications. It is a serious concern that in the midst of the opioid overdose epidemic there is a growing presence of kratom use in the U.S. population with a largely unregulated status.

## 1 Background

*Mitragyna speciosa* is an herbal leaf native to evergreen trees in southeast Asia that is more commonly known as kratom. Kratom is classified as a new psychoactive substance (NPS). Historically, kratom has been used as an herbal medicine, but its opioid properties and stimulant-like effects have become increasingly used for recreation, and it has been noted for its significant abuse potential worldwide (1). There are various descriptions on the safety of kratom, with some reporting that it is a safe and legal psychoactive substance that can modulate mood and pain and even function as an alternative medication-assisted treatment for opioid use disorder (1, 2). However, other reports show that kratom’s harm potential is high, and its current unregulated use in the United States is likely dangerous (1–3). In 2019, the United States Food and Drug Administration (FDA) issued a public statement discouraging the use of kratom and explicitly pointing out that there is not currently enough evidence that kratom is an effective treatment for any condition (4). Still, the legal status and regulation of kratom varies widely from state to state.

The risk of adverse reactions including death regarding kratom use and the combination of kratom with other substances, both illicit and licit, remains a serious concern for clinicians who seek to provide education to their kratom-using patients. Between 2011 and 2017, more than 200,000 people died from opioid-related overdoses in the United States (5). Post et al. analyzed data from the National Poison Data System also between the years 2011–2017 and found that 11 deaths were associated with kratom exposure, including two that occurred with exposure only to kratom (6). In this manuscript, we report an additional four cases of opioid overdose deaths with qualitative kratom findings found on the coroner reports.

## 2 Methods

This study investigates coroner reports from Kern County, California for the year 2020. Notably, we also reviewed reports from the years 2018 and 2019, but there were no kratom-associated deaths in those years. Information provided included mode of death, cause of death, sex, age, race/ethnicity, and qualitative substances found post-mortem. Toxicological detection in Kern county is provided by use of gas or liquid chromatography with mass spectrometry; kratom use was identified by qualitative mitragynine finding, other metabolites of kratom were not identified (7). Subject names, medical records, date of birth, government identifiers, or any other potential specific identifiers were not provided to the investigators. No measurement of toxicological substances was available (including detection limits, sensitivity and specificity, quality control measures, etc.) from the coroner report provided to the study investigators. We approached our local institutional review board for study approval and were advised that the review was not necessary to proceed because our dataset involves only de-identified decedent health information. For this type of health information, in accordance with the journal guidelines (Frontiers in Psychiatry), we converted exact ages into 5-year age ranges.

A database was created using coroner reports resulting in 773 total subjects. The coroner’s office reports the mode of death by the following categories: accidental (*n* = 478), homicide (*n* = 109), suicide (*n* = 47), natural (*n* = 93), and undetermined (*n* = 46). In addition to mode of death, the coroner’s office also reports the medical cause of death, which included a range of clinical conditions including toxicity (overdose), trauma, cardiovascular, metabolic, and more (but not relevant to the current study).

A broad range of substances (including an array of metabolites) were qualitatively tested for and found in the coroner reports. For the purposes of this study, these were consolidated into the following categories: cannabis, amphetamines, mitragynine (kratom), fentanyl, other opioids, benzodiazepines, antihistamines, antipsychotics, and antidepressants. Fentanyl was distinguished from other opioids due to its current rising use and associated accidental overdoses. Quantitative test results were not available to us.

## 3 Results

Four subjects (0.52% of the entire coroner report database for 2020) had mitragynine (kratom) exposure and were included in this study. Each of the mitragynine-associated cases had a mode of death of “accident” and a cause of death of “toxicity” (overdose). There were 214 opioid-associated accidental overdoses in the year 2020 for Kern County, with 4 (1.9%) of these having specific toxicology for mitragynine. Mitragynine exposure never occurred in isolation in these cases; when mitragynine was found in the coroner’s reports, there was a comorbid substance that likely contributed to the death of the subject. All mitragynine-exposed subjects were white, mean age was 39 years, 3 were male and 1 was female (Table 1). The most common substance identified concurrently with mitragynine was fentanyl (75%), but other opioids, benzodiazepines, cannabis, and psychiatric medications were identified. One case did not have a concurrent fentanyl finding (case #1), but the following medications were identified: gabapentin, alprazolam, clonidine, buspirone, and cyclobenzaprine.

**Table 1:** Sociodemographic information and substances identified in the coroner’s report for each case.

Case #	Age range	Race	Sex	Mitragynine (Kratom)	Fentanyl	Other opioid	Benzodiazepine	Cannabis	Antihistamine	Antidepressant	Antipsychotic	Antiepileptic
1	35–40	White	Female	+	−	−	+	−	+	+	+	+
2	45–50	White	Male	+	+	+	−	−	+	−	−	−
3	25–30	White	Male	+	+	−	−	+	−	−	−	−
4	45–50	White	Male	+	+	−	−	−	+	−	−	−

In all cases race was reported as white, the mode of death was accidental, cause of death was toxicity. Five-year age ranges used in place of exact age to comply with *Frontiers in Psychiatry* guidelines for decedent health information.

## 4 Discussion

The results of this brief report provide insight into the role kratom use has on modulating the risk of opioid overdose death in the context of concurrent use with other substances. The combined use of kratom (mitragynine) with other opioids (such as fentanyl) is known to increase risk of overdose (cases 2–4). However, an additional finding from this study implicates that mitragynine use combined with benzodiazepine use (and other non-opioid medications seen in case 1), also increases the risk of opioid overdose. The combination of opioids with benzodiazepines is a known risk factor for opioid toxicity (8), and we urge clinicians to consider this risk for their kratom using patients who are prescribed benzodiazepines.

There are limitations to the findings of this study, including limited clinical information about each case. We were not able to obtain quantitative values for the substances found in descendants, which limits our ability to interpret the individual and combined role that each substance contributed to each opioid overdose death. Further, we were not able to correlate if the use of each substance was from intended use or from adulterated products. Finally, there is a lack of studies that detail the interaction of mitragynine and its metabolites among psychiatric medications.

In 2019, Henningfield et al. estimated that the risk of overdose death is over 1,000 times greater for opioids than for mitragynine alone (9). The average lethal dose of fentanyl is estimated at serum concentrations of 0.025 μg/mL (0.005–0.027 μg/mL) (10), while the lethal dose of kratom is 0.398 mg/L (range = 0.0035–0.890 mg/L); however, the kratom estimates are from a very small sample sizes study (*n* = 3) (11).

Adzrago et al. analyzed the 2020 NSDUH data and concluded that kratom use was more frequently associated with individuals who had cannabis, opioid, and alcohol use disorders, as well as those with major depressive disorder (12, 13). Kratom use appears to be on the rise in the United States, with data from the 2019 National Survey on Drug Use and Health (NSDUH) estimating 11.1% lifetime and 6.7% past-year kratom use (14). Accidental overdoses with mitragynine toxicology are rare, but as use of kratom continues to increase in the United States, there is a need to monitor the role kratom may have in modulating risk of opioid toxicity. In this report, our county also observed increasing kratom use over time, with no kratom associated deaths reported for 2018 and 2019, but then 4 reported in 2020.

Kratom-associated fatalities have also been reported when use was combined with carisoprodol, modafinil, propylhexedrine, diphenhydramine, acetaminophen, and caffeine (1, 15, 16). There are also other reports of non-lethal but serious adverse reactions when kratom has been used with other substances. One case report described an individual self-treating opioid withdrawal with kratom developing a seizure when use was combined with modafinil (17). Independent kratom use has been reported to induce various medical and psychiatric problems, including seizures (18, 19), liver injury (20), hyperkalemia (21), acute respiratory distress syndrome (22), and psychosis (23, 24).

There are further complications when attempting to determine lethal and non-lethal risks associated with kratom use. This includes significant variations in kratom preparation, strains of kratom, and the relative strength of each preparation. Chemically, kratom’s mechanism of action is largely attributed to its alkaloid components of mitragynine and 7-hydroxymitragynine, which are full agonists of μ- and δ-opioid receptors (1). Mitragynine has approximately 13 times the potency of morphine for its opioid effects (1, 2), and 7-hydroxymitragynine has both potent CNS stimulant and depressant effects (1, 2). The leaves of the kratom plants with red veins tend to provide more analgesia compared to the leaves with white or green veins, which tend to be more stimulating and mood-enhancing (1). Still, the dose of each strain can be modified to achieve different effects, with low doses (under 5 g) providing stimulant effects, and higher doses (5–15 mg) providing opioid-like effects (1).

## 5 Conclusion

This brief report describes 4 cases of opioid toxicity fatalities with concurrent mitragynine toxicity. The toxicology findings of this report suggest that when kratom is used with other opioids (such as fentanyl) there is likely increased risk of a fatal overdose. This risk may also be increased by other medications such as benzodiazepines and other psychiatric medications. It is a serious concern that in the midst of the opioid overdose epidemic there is a growing presence of kratom use in the U.S. population. In many states kratom is legal for purchase, easily accessible, and is unregulated in terms of content and possible contamination by other substances. This clearly introduces an increased level of risk that needs to be addressed by education of the public and health care system.

## Data availability statement

The original contributions presented in the study are included in the article/supplementary material, further inquiries can be directed to the corresponding author.

## Ethics statement

The requirement of ethical approval was waived by the Kern Medical Institutional Review Board for the studies involving humans because the IRB advised that review was not necessary due to the data involving only de-identified public health information of deceased individuals. The studies were conducted in accordance with the local legislation and institutional requirements. The ethics committee/institutional review board also waived the requirement of written informed consent for participation from the participants or the participants’ legal guardians/next of kin because the IRB advised that review was not necessary due to the data involving only de-identified public health information of deceased individuals.

## Author contributions

TT: Conceptualization, Formal analysis, Validation, Writing – original draft, Data curation, Investigation, Methodology. KP: Data curation, Validation, Writing – review & editing. NN: Data curation, Validation, Writing – review & editing. MS: Supervision, Validation, Writing – original draft. RP: Supervision, Validation, Writing – review & editing. DW: Supervision, Validation, Writing – review & editing.
